# Glucagon-Like Peptide-1 (GLP-1) Receptor Agonist Use and Inflammatory Markers Among U.S. Adults: A National Health and Nutrition Examination Survey (NHANES)-Based Analysis

**DOI:** 10.7759/cureus.90964

**Published:** 2025-08-25

**Authors:** Sharokeen Youkhana, Seun S Odusanmi, Bruno K Ihuchukwu, Mathew Ezekiel, Edidiong Enyeneokpon, Christina Cumaaran, Emmanuel O Akpamgbo, Okelue E Okobi

**Affiliations:** 1 General Medicine, Hawler Medical University, Erbil, IRQ; 2 Clinical Hematology, University Hospital of Leicester, Leicester, GBR; 3 General Medicine, St Mary's Hospital, Isle of Wight NHS Trust, Newport, GBR; 4 General Medicine, Texila American University, Georgetown, GUY; 5 Acute Medicine, Pilgrim Hospital, Boston, GBR; 6 Family and Community Medicine, American International School of Medicine, Georgetown, GUY; 7 Family Medicine, Emergency Medicine, Obstetrics and Gynecology, Abia State University, Uturu, NGA; 8 Family Medicine, Larkin Community Hospital, Palm Springs Campus, Miami, USA

**Keywords:** c-reactive protein, cross-sectional analysis, glp-1 receptor agonists, nhanes, systemic inflammation, type 2 diabetes mellitus

## Abstract

Background: Systemic inflammation plays a critical role in the development of obesity-related complications, type 2 diabetes mellitus (T2DM), and cardiovascular disease. Glucagon-like peptide-1 receptor agonists (GLP-1RAs) are widely used for glycemic control and weight management, with emerging evidence suggesting potential anti-inflammatory effects.

Objective: To examine the association between GLP-1RA use and systemic inflammation, measured by high-sensitivity C-reactive protein (hs-CRP), in a nationally representative sample of U.S. adults.

Methods: We conducted a cross-sectional analysis using National Health and Nutrition Examination Survey (NHANES) 2005-2010 data. Survey-weighted linear regression models were used to assess the relationship between GLP-1 use and log-transformed CRP, adjusting for demographic, socioeconomic, and clinical factors.

Results: GLP-1 users had significantly higher CRP levels in unadjusted models (β=1.081, p<0.01). However, after adjusting for age, gender, race/ethnicity, income, body mass index (BMI), diabetes, glycated hemoglobin (HbA1c), and smoking, the association was no longer statistically significant (β=0.323, p=0.38). Clinical factors accounted for most of the variance.

Conclusion: In this cross-sectional, population-based analysis, GLP-1RA use was not independently associated with lower systemic inflammation after accounting for demographic and clinical confounders. The higher hs-CRP observed among GLP-1 users in unadjusted models is most consistent with confounding by indication and greater cardiometabolic disease burden among treated individuals rather than a direct pro-inflammatory effect of the medications. Longitudinal studies with larger contemporary samples (including newer GLP-1 agents) and repeated inflammatory measures are needed to clarify whether GLP-1 therapies exert direct anti-inflammatory effects beyond their metabolic benefits.

## Introduction

Currently, obesity, type 2 diabetes mellitus (T2DM), and cardiovascular disease are among the common health issues that affect the U.S. population in millions and cause high morbidity and even death [1,2]. Systemic inflammation, a low-grade, persistent inflammatory condition, is one of the common physiologic strands shared by these chronic diseases that leads to insulin resistance, atherosclerosis, and various metabolic imbalances [3]. Systemic inflammation promotes insulin resistance and atherogenesis through several well-described mechanisms: pro-inflammatory cytokines (such as tumor necrosis factor-α (TNF-α) and interleukin-6 (IL-6)) induce serine phosphorylation of insulin receptor substrate proteins and impair downstream insulin signaling, promote lipolysis and ectopic lipid deposition, and increase oxidative stress; concurrently, inflammation drives endothelial dysfunction, monocyte recruitment, macrophage foam cell formation, and matrix remodeling that accelerate plaque formation and instability. These cellular and molecular pathways collectively link chronic low-grade inflammation to impaired glucose homeostasis and atherosclerotic cardiovascular disease [3].

One of the acute-phase proteins produced in the liver is C-reactive protein (CRP), which is well known as a solid biomarker of systemic inflammation [4]. There has been an independent connection between an elevated level of CRP and a higher risk of cardiovascular events, worsened glycemic control, and all-cause mortality [5]. Because of this, treatment modalities that would successfully mitigate broad systemic inflammation may hold wide implications for the overall metabolic and cardiovascular health [6].

Glucagon-like peptide-1 receptor agonists (GLP-1RAs) are a group of drugs that have attracted a lot of interest in recent years as potential blockbuster therapeutics initially aimed at the treatment of T2DM, but are increasingly being noticed in terms of the multiple levels they can positively influence [7,8]. These are injectable drugs that act like endogenous GLP-1 in order to increase glucose-stimulated insulin secretion, decrease the release of glucagon, and reduce smooth emptying [9]. Notably, in addition to their anti-hyperglycemic properties, GLP-1RAs have also been found to induce weight loss, as well as reduce lipid levels and lead to valuable cardiovascular protection, a factor that has allowed them to become a key pillar in the treatment of both obesity and diabetes [10,11]. Beyond glucose lowering, GLP-1RAs confer multiple additional benefits relevant to obesity and cardiometabolic health, including reductions in body weight and visceral adiposity through appetite suppression and delayed gastric emptying, modest improvements in blood pressure and lipid profiles, and demonstrated reductions in major adverse cardiovascular events in several outcome trials; some data also suggest renal-protective effects and favorable changes in adipose tissue inflammation and endothelial function [7,10,11].

Recent evidence points to the possible anti-inflammatory property of GLP-1RAs as well [12]. During animal experiments and small-scale clinical tests, a decrease in indicators of inflammatory activity, including CRP, IL-6, and TNF-α, was observed in patients under the influence of GLP-1RAs [13,14]. Among the postulated mechanisms of action, there are weight reduction, glycemic control, modulation of macrophage activation, and attenuation of oxidative stress [15]. Nevertheless, even based on these encouraging results, population-based data on the issue of the use of GLP-1RAs and inflammation, in particular, relating to the general adult population in the United States, has yet to be done [16].

It is clinically relevant to understand the relation between the use of GLP-1RAs and reduced CRP levels in real-world patients [17]. If GLP-1RAs reduce systemic inflammation independent of improvements in glycemic control, this would indicate potential disease-modifying effects beyond glucose lowering and have important implications for cardiovascular risk reduction and clinical decision-making [18]. It can also impact prescribing decisions and guidelines and create additional opportunities for cardiovascular risk management in people with obesity and T2DM [19].

The National Health and Nutrition Examination Survey (NHANES) provides a special chance to examine this relation among a large, varied, and nationally representative group of adults in the U.S. NHANES has all-encompassing data on health conditions, pharmacological intake, laboratory indicators, and sociodemographic characteristics that enable a detailed study of the connection between pharmacologic activities and separate outcomes among numerous groups of population members [20]. The main objective of the study is to investigate the association between GLP-1RA use and systemic inflammation (CRP) in a representative sample of U.S. adults.

## Materials and methods

Study design and data source

This study utilized publicly available data from the NHANES, a nationally representative cross-sectional survey conducted by the National Center for Health Statistics (NCHS). Data from the 2005 and 2010 survey cycles were selected for analysis. This time frame was chosen for two primary reasons: first, high-sensitivity C-reactive protein (hs-CRP), the primary biomarker of systemic inflammation in this study, was consistently measured and available during these years; and second, GLP-1RAs, specifically exenatide (Lexicon Plus drug code: d05529) and liraglutide (d07466), were Food and Drug Administration (FDA) approved and included in the NHANES prescription medication dataset beginning in 2005 and 2010, respectively. The NHANES dataset provides detailed information on prescription medication use, laboratory biomarkers, sociodemographic characteristics, and health-related behaviors among the non-institutionalized U.S. population [21].

Study population

Eligible participants included adults aged 18 years and older from the NHANES 2005-2010 cycles who had available data on prescription medication use and hs-CRP measurements. Both GLP-1RA users and non-users were included to allow for comparative analysis. Individuals with missing values for key covariates were retained to maximize power, given the small number of GLP-1 users.

Exposure variable: glucagon-like peptide-1 receptor agonist use

The primary exposure of interest was self-reported use of GLP-1RAs. Medication use was determined based on responses to the NHANES prescription medication questionnaire, where participants reported all prescription drugs taken in the past 30 days. Medications were categorized using the Lexicon Plus drug classification system. Participants were classified as GLP-1RA users if they reported any of the following medications: exenatide or liraglutide, which were present during the study period.

Outcome variable: inflammatory marker

The primary outcome was systemic inflammation, assessed using hs-CRP levels, measured in milligrams per liter (mg/L). Hs-CRP is a validated biomarker of systemic low-grade inflammation and was measured using latex-enhanced nephelometry. Participants with hs-CRP levels above 10 mg/L were excluded to minimize the influence of acute infections or inflammatory conditions unrelated to metabolic health.

Covariates

Potential confounders included demographic and socioeconomic characteristics (age, sex, race/ethnicity, family poverty income ratio, and education level), clinical factors (body mass index (BMI), diabetes status, and smoking status), and the laboratory marker glycated hemoglobin (HbA1c). These variables were selected based on prior literature linking them to both GLP-1 use and inflammation [3,5,13,17]. BMI was calculated from measured height and weight, and diabetes status was determined from self-reported physician diagnosis and/or use of antidiabetic medications. Smoking status was classified as current or never smoker. We acknowledge that additional factors, including dietary patterns, physical activity, duration of GLP-1 therapy, and medication adherence, may also act as potential confounders. Although NHANES collects dietary recall and physical activity data in some cycles, these measures are variably collected across cycles, prone to recall and measurement error, and do not reliably align with the 30-day medication reporting window; furthermore, NHANES prescription data capture reported use in the prior 30 days but do not provide robust information on treatment duration or adherence for GLP-1 agents. Therefore, these variables were not included in the primary models, and residual confounding by these unmeasured factors is possible; this limitation is addressed further in the Discussion and Limitations sections.

Missing data handling

Given the small number of participants exposed to GLP-1RAs in the sample (n=21), observations with missing values were not excluded through listwise deletion to preserve analytical power. Most covariates had low levels of missingness, with the proportion of missing data remaining below 10% for all key variables (e.g., education: 3.3%, income-to-poverty ratio: 8.4%, BMI: 4.9%, HbA1c: 9.0%). These levels are generally considered acceptable for complete case analysis in epidemiological studies. Moreover, due to the application of NHANES’ complex survey design and weighting procedures, conventional multiple imputation methods were not used, as they are not directly supported within the survey analysis framework in Stata. As such, missing values were retained, and no imputation was performed. This decision balances the need for valid population estimates while minimizing the loss of rare exposures.

Statistical analysis

All analyses incorporated the complex, multistage sampling design of the NHANES using survey weights, masked variance strata, and primary sampling units, as recommended by the NCHS. The Mobile Examination Center (MEC) examination weights were applied, given the inclusion of laboratory data. Descriptive statistics were computed to compare characteristics between GLP-1RA users and non-users. Continuous variables were summarized as survey-weighted means and standard deviations, while categorical variables were reported as frequencies and weighted percentages. Group differences were assessed using survey-adjusted t-tests for continuous variables and F-tests from adjusted chi-square procedures for categorical variables.

To evaluate the association between GLP-1RA use and systemic inflammation, we modeled log-transformed hs-CRP levels using survey-weighted linear regression. Three models were specified: Model 1 was unadjusted; Model 2 adjusted for demographic and socioeconomic variables (age, gender, race/ethnicity, education level, and income-to-poverty ratio); and Model 3 further adjusted for clinical and behavioral covariates, including BMI, diabetes status, HbA1c, and current smoking. Adjusted beta coefficients and standard errors were reported. A two-sided p-value < 0.05 was considered statistically significant. All analyses were conducted using Stata version 18.0 (StataCorp LLC, College Station, Texas, U.S.).

Ethical considerations

This study was based on secondary analysis of publicly available data from the NHANES, which is conducted by the NCHS, a division of the Centers for Disease Control and Prevention (CDC). All NHANES protocols are approved by the NCHS Research Ethics Review Board, and informed consent was obtained from all participants at the time of data collection. The current analysis involved de-identified data and did not require additional ethical approval or informed consent. In accordance with the guidelines for research using publicly available datasets, this study was deemed exempt from institutional review board (IRB) oversight.

## Results

Table 1 below presents the survey-weighted baseline characteristics of adults included in the NHANES 2005-2010 cycles, stratified by use of GLP-1RAs. Weighted estimates reflect national population distributions, and comparisons were made using design-based t-tests for continuous variables and F-tests for categorical variables to account for NHANES’ complex sampling design. The exposed group represents an estimated 289513 GLP-1 users in the U.S. population during this period, compared to 217993702 non-users.

**Table 1:** Survey-weighted baseline characteristics of U.S. adults by GLP-1RA use, NHANES 2005-2010 ^*^p<0.05. ^**^p<0.01. ^***^p<0.001. All values are survey-weighted national estimates. Categorical variables are reported as unweighted n (weighted %). Continuous variables are reported as weighted mean±SD. Comparisons are based on design-adjusted t-tests or F-tests using NHANES survey weights. GLP-1RA: glucagon-like peptide-1 receptor agonist; SD: standard deviation; HbA1c: glycohemoglobin; CRP: C-reactive protein; -: intentionally left blank; NHANES: National Health and Nutrition Examination Survey

Variable	GLP-1 users (N=289513)	GLP-1 non-users (N=217993702)	Design-based F/t-test	P-value
High-sensitivity CRP (mean±SD)	-0.69±0.97	-1.76±1.30	t=-3.09	0.003
Age (in years), (mean±SD)	52.72±8.21	46.24±16.83	t=-2.63	0.01
Gender(%)	-	-	F=0.36	0.551
Male	118836 (0.11%)	105235319 (99.89%)	-	-
Female	170677 (0.15%)	112758383 (99.85%)	-	-
Race/ethnicity (%)	-	-	F=0.99	0.392
Mexican American	9632 (0.05%)	18325353 (99.95%)	-	-
Other Hispanic	6510 (0.07%)	9772243 (99.93%)	-	-
Non-Hispanic White	230007 (0.15%)	151367030 (99.85%)	-	-
Non-Hispanic Black	43362 (0.17%)	25043972 (99.83%)	-	-
Other race	0.00	13485102 (100%)	-	-
Education level (adults 20+), (%)	-	-	F=0.82	0.483
Less than 9^th^ grade	12591 (0.09%)	14117953 (99.91%)	-	-
9-11^th^ grade	6162 (0.02%)	26878190 (99.98%)	-	-
High school grad/GED	64231 (0.12%)	52345211 (99.88%)	-	-
Some college or AA degree	135560 (0.21%)	64531223 (99.79%)	-	-
College graduate or above	70967 (0.13%)	56311443 (99.87%)	-	-
Family poverty income ratio	3.82±1.16	3.04±1.63	t=-2.21	0.03
BMI (kg/m^2^)	36.55±5.19	28.61±6.71	t=-4.70	p<0.001
The doctor told you that you have diabetes (%)	-	-	F=68.12	p<0.001
Yes	256396 (1.44%)	17530610 (98.56%)	-	-
No	33117 (0.02%)	196695732 (99.98%)	-	-
HbA1c (%), (mean±SD)	7.42±1.72	5.53±0.84	t=-4.06	p<0.001
Smoking status (%)	-	-	F=12.41	p<0.001
Not a current smoker	283622 (0.17%)	16647167 (99.83%)	-	-
Current smoker	5891 (0.01%)	47905527 (99.99%)	-	-

Table 1 summarizes the baseline characteristics of participants by GLP-1RA use. GLP-1 users were significantly older, with a mean age of 52.72±8.21 years compared to 46.24±16.83 years in non-users (p=0.01). Mean BMI was markedly higher in GLP-1 users (36.55±5.19 kg/m² vs. 28.61±6.71 kg/m²; p<0.001), consistent with the medication's indication for obesity and type 2 diabetes management. HbA1c levels were also higher in the exposed group (7.42±1.72 vs. 5.53±0.84, p<0.001), and the prevalence of self-reported diabetes was significantly greater among GLP-1 users 256396 (1.44%) vs. 33117 (0.02%).

Notably, the average log-transformed CRP value was significantly lower among GLP-1 users compared to non-users (-0.69±0.97 vs. -1.76±1.30, p=0.003), suggesting potential anti-inflammatory effects associated with GLP-1 therapy. There were no significant differences in gender, race/ethnicity, or educational attainment between the two groups. However, GLP-1 users had a significantly higher family income-to-poverty ratio (p=0.03), indicating possible socioeconomic differences. The prevalence of current smoking was significantly lower among GLP-1 users 5891(0.01%) vs. 283622(0.17%), p<0.001), which may partly account for differences in CRP levels.

Table 2 presents the results of three sequential linear regression models assessing the association between GLP-1RA use and systemic inflammation, measured by log-transformed high-sensitivity CRP. Model 1 is unadjusted, Model 2 adjusts for demographic and socioeconomic characteristics, and Model 3 incorporates clinical and behavioral covariates. All models account for the complex sampling design of NHANES through survey weighting. The objective was to determine whether GLP-1 use independently predicts lower levels of systemic inflammation among U.S. adults.

**Table 2:** Survey-weighted multivariate linear regression models predicting log-transformed CRP levels by GLP-1 use and covariates ^*^p < 0.05. ^**^p < 0.01. ^***^p < 0.001. All regression models are survey-weighted and account for the NHANES complex sampling design using strata, clusters, and MEC examination weights. Model 1 is unadjusted, Model 2 adjusts for age, gender, race/ethnicity, education level, and family income-to-poverty ratio, and Model 3 additionally includes BMI, diabetes diagnosis, HbA1c level, and smoking status. Coefficients represent beta estimates for predictors of log-transformed CRP. Standard errors are reported in parentheses. NA: variable not included in this model; GLP-1: glucagon-like peptide-1; CRP: C-reactive protein; BMI: body mass index; HbA1c: glycohemoglobin; -: intentionally left blank; SE: standard error

Predictors	Model 1	Model 2	Model 3
GLP-1 use	1.081^**^	1.015^**^	0.323
-	(SE0.341)	(0.346)	(0.370)
Age (in years)	NA	0.010^***^	0.007^***^
-	-	(0.001)	(0.001)
Gender (female)	NA	0.286^***^	0.325^***^
-	-	(0.025)	(0.024)
Race/ethnicity	-	-	-
Other Hispanic	NA	-0.136^*^	-0.118
-	-	(0.063)	(0.061)
Non-Hispanic White	NA	-0.191^***^	-0.146^**^
-	-	(0.053)	(0.042)
Non-Hispanic Black	NA	0.033	-0.123^*^
-	-	(0.054)	(0.048)
Other race	NA	-0.502^***^	-0.315^***^
-	-	(0.077)	(0.062)
Education level (adults 20+)	-	-	-
9-11^th^ grade	NA	0.092	0.009
-	-	(0.052)	(0.041)
High school grad/GED	NA	0.077	0.014
-	-	(0.053)	(0.048)
Some college or AA degree	NA	0.011	-0.041
-	-	(0.043)	(0.042)
College graduate or above	NA	-0.207^**^	-0.090
-	-	(0.059)	(0.048)
Family poverty income ratio	NA	-0.039^***^	-0.027^**^
-	-	(0.010)	(0.008)
BMI (kg/m^2^)	NA	NA	0.087^***^
-	-	-	(0.002)
Doctor told you have diabetes	NA	NA	0.173^**^
-	-	-	(0.050)
HbA1c (%)	NA	NA	0.114^***^
-	-	-	(0.016)
Current smoker	NA	NA	0.300^***^
-	-	-	(0.029)
Constant	-1.757^***^	-2.057^***^	-5.532^***^
-	(0.021)	(0.062)	(0.165)
Observations	16689	14949	14708
R^2^	0.001	0.056	0.265

In the unadjusted model (Model 1), GLP-1RA use was significantly associated with higher log-transformed CRP levels (β=1.081, p<0.01), suggesting that GLP-1 users exhibited elevated systemic inflammation compared to non-users. This association persisted in Model 2 (β=1.015, p<0.01) after adjusting for age, gender, race/ethnicity, education, and income. However, upon inclusion of clinical and behavioral factors in Model 3, the association between GLP-1 use and CRP was no longer statistically significant (β=0.323, p=0.38). This decline indicates that the initial relationship was likely confounded by health-related characteristics common among GLP-1 users such as higher body mass index, diabetes status, glycemic control, and smoking behavior.

Among the covariates, age was positively associated with CRP in all adjusted models (Model 2: β=0.010; Model 3: β=0.007; both p<0.001), indicating a modest rise in inflammation with increasing age. Female participants had significantly higher CRP levels than males (Model 3: β=0.325, p<0.001). Higher BMI (β=0.087, p<0.001), current smoking (β=0.300, p<0.001), diabetes diagnosis (β=0.173, p<0.01), and elevated HbA1c (β=0.114, p<0.001) were all significantly associated with higher CRP levels, underscoring their role as key drivers of systemic inflammation.

The R-squared values increased across the models, from 0.001 in the unadjusted model to 0.265 in the fully adjusted model, indicating improved explanatory power as more relevant variables were included. Overall, while the unadjusted models suggested a strong link between GLP-1 use and inflammation, the association was rendered non-significant after accounting for demographic and clinical factors, highlighting the influence of underlying comorbidities and risk profiles among GLP-1 users.

## Discussion

GLP-1 RAs exert their anti-inflammatory effects through multiple mechanisms, including changing cytokine production, directly interacting with immune cells, and influencing toll-like receptor (TLR) signaling pathways. Previous studies have consistently demonstrated that GLP-1RAs can lower systemic inflammation markers and enhance endothelial health [12]. Although earlier research established the anti-inflammatory role of GLP-1 broadly, our detailed analysis highlights specific cellular interactions and signaling pathways, providing a deeper understanding of how GLP-1 benefits go beyond simple glycemic regulation [2]. In this nationally representative, survey-weighted analysis of U.S. adults from the 2005-2010 NHANES cycles, we investigated the association between GLP-1RA use and systemic inflammation, as measured by hs-CRP. Our findings revealed that although GLP-1 users initially exhibited higher levels of CRP in unadjusted models, the association was no longer statistically significant after accounting for demographic, metabolic, and behavioral covariates. These results suggest that the observed differences in CRP are likely attributable to the underlying clinical characteristics of GLP-1 users, namely, higher rates of obesity, diabetes, and poorer glycemic control, rather than a direct anti-inflammatory effect of the medication.

The unadjusted model showed a significant positive association between GLP-1 use and log-transformed CRP levels. This effect persisted when adjusting for demographic and socioeconomic variables, but diminished and lost significance in the fully adjusted model. This pattern emphasizes the importance of accounting for clinical covariates such as BMI, diabetes status, HbA1c, and smoking, each of which independently correlated with elevated CRP levels in our analysis. These findings are consistent with prior studies showing that obesity, hyperglycemia, and tobacco use are key drivers of chronic low-grade inflammation [3,5].

Importantly, the initial higher CRP levels among GLP-1 users likely reflect confounding by indication. In real-world settings, GLP-1RAs are typically prescribed to individuals with advanced type 2 diabetes or obesity, both of which are associated with heightened inflammatory states [1,7,11]. Our study affirms this clinical pattern, as GLP-1 users had significantly higher mean BMI (36.55±5.19 kg/m² vs. 28.61±6.71 kg/m²) and HbA1c levels (7.42±1.72% vs. 5.53±0.84%) compared to non-users. Once these covariates were introduced into the model, the independent association between GLP-1 use and CRP was no longer statistically significant, suggesting that the elevated inflammation was more closely linked to metabolic burden than medication use itself.

Contrary to early experimental and small-scale clinical studies suggesting anti-inflammatory effects of GLP-1RAs [12-14], our findings do not support a significant association in a population-based context. While GLP-1 therapies have shown reductions in inflammatory biomarkers like CRP, IL-6, and TNF-α in controlled settings, these effects may be confounded by weight loss, improved glycemic control, or reductions in visceral adiposity mechanisms that take time to manifest and are often difficult to isolate in cross-sectional studies [13,15]. Further, our findings suggest that the disease itself drives the inflammatory responses we observed, standing in contrast to earlier studies that often highlighted the anti-inflammatory advantages of GLP-1s. Previous research by Ferhatbegović et al. in 2023 has shown that GLP-1s have direct anti-inflammatory effects, helping to improve cardiovascular health and metabolic outcomes [11]. This earlier evidence indicated that GLP-1s could actively reduce the inflammatory processes associated with obesity and diabetes [2]. These divergent findings underscore the need for cautious interpretation: controlled trials and mechanistic studies document anti-inflammatory effects of GLP-1RAs, but such population-based, cross-sectional analyses may fail to detect these effects because they capture heterogeneous, treatment-indicated populations with greater baseline metabolic burden. While randomized and mechanistic evidence supports anti-inflammatory actions of GLP-1 therapies, persistent inflammation in some patients likely reflects underlying disease severity or duration that may not be fully reversible by pharmacologic therapy alone. 

Limitations and strengths of the study

This study is limited by the small number of GLP-1 users in the 2005-2010 NHANES dataset, which reflects the early adoption phase of exenatide and liraglutide following their FDA approval. This limited sample size reduces statistical power and may mask true associations. Additionally, the analysis grouped all GLP-1 users together without stratifying by dosage, duration of use, or specific GLP-1 agent; different agents, doses, or treatment durations could plausibly exert varying anti-inflammatory effects, and lack of these exposure details may have obscured heterogeneity in inflammatory outcomes. Additionally, the cross-sectional design prevents any conclusions about causality or temporal direction between GLP-1 use and CRP levels. Data on prescription drug use are self-reported, which may introduce recall bias; however, this bias is partially mitigated by verifying medication containers. The analysis was also limited to data up to 2010, as hs-CRP was not available in later NHANES cycles. There is additionally potential for geographic or regional underrepresentation bias, particularly for transient or rural populations, since the NHANES study visited specific communities in rotating cycles. Also, the fact that participation is voluntary could have led to nonresponse bias, skewing prevalence estimates, especially among low-income, non-English-speaking, or undocumented populations. Logistical constraints are another limitation contributing to sampling bias. Scheduling and time limitations may have arisen because some individuals are shift workers, caregivers, or have restrictive schedules. The reliance on mobile exam centers, while they were standardized and well equipped, had limited availability, and access may still pose a barrier for participants who live farther away or lack transportation, even with provided transport services. Household-based sampling ties into this, as individuals in institutional settings (e.g., nursing homes, correctional facilities, and military barracks) are excluded, despite their unique health profiles, limiting generalizability. Furthermore, environmental and temporal confounders are yet another limitation. Since data collection is spread over different seasons and regions, environmental factors (e.g., seasonal diet changes, pollen exposure, and flu season) may influence health measures and introduce variability that complicates interpretation. The model 3 covariates, which interpreted the data after adjusting for potential confounders, did not include these additional factors.

Despite these limitations, the study is strengthened by the use of a nationally representative sample, standardized laboratory testing, which, together with NHANES’s complex sampling design and weighting, enhances the generalizability of the findings, and real-world data on medication use. It offers a rare snapshot of early GLP-1 use and inflammation profiles at the population level. Future research should use more recent NHANES data or longitudinal cohorts where GLP-1 use is more widespread and better documented. Larger sample sizes and newer GLP-1 agents (e.g., semaglutide) should be included to better assess associations with inflammation. Studies using prospective designs are also encouraged to clarify causal relationships.

## Conclusions

In this nationally representative analysis of U.S. adults, GLP-1RA use was not independently associated with lower levels of systemic inflammation after adjusting for demographic and clinical factors. The initially observed differences in CRP were largely explained by underlying metabolic and behavioral characteristics common among GLP-1 users. Because this study is cross-sectional and observational, causality cannot be inferred; despite the use of survey-weighted models and adjustment for major confounders, residual confounding (for example, by diet, physical activity, duration of therapy, and medication adherence) and the small number of GLP-1 users in the sample may limit the strength of the conclusions and our ability to detect a true medication effect. Therefore, we caution against attributing direct anti- or pro-inflammatory effects to GLP-1 therapies on the basis of these data alone. Longitudinal studies with larger, contemporary samples (including newer GLP-1 agents) and repeated inflammatory measures are needed to determine whether GLP-1 therapies exert direct anti-inflammatory effects beyond their established metabolic benefits.
